# THE EFFECT OF MOOD ON SELF-REPORTED COGNITION FOR INDIVIDUALS WITH HIV

**DOI:** 10.1093/geroni/igac059.2072

**Published:** 2022-12-20

**Authors:** Emilee Ertle, Darby Simon, Jax-Patrick Vogt, Benjamin Mast, Shirish Barve

**Affiliations:** University of Louisville, Louisville, Kentucky, United States; University of louisville, Louisville, Kentucky, United States; University of Louisville, Louisville, Kentucky, United States; University of Louisville, Louisville, Kentucky, United States; University of Louisville, Louisville, Kentucky, United States

## Abstract

Older adults with HIV may experience cognitive changes. The Patient Assessment of Own Functioning Inventory (PAOFI) is a self-report measure of cognitive abilities with memory, language, and cognitive/intellectual functions domains. Self-reported cognition can be influenced by many factors. This study examined the relationship between demographics, performance-based cognitive tests, mood, and self-reported cognition in a sample of 23 HIV-positive individuals (mean age=56.7). The PAOFI memory domain positively correlated with depression (PHQ-9; r=.64, p=.001) and anxiety (GAD-7; r=.48, p=.019), but not tests of cognition. The PAOFI cognitive/intellectual functions domain also positively correlated with the PHQ-9 (r=.65, p=.001) and the GAD-7 (r=.66, p=.001), but not cognitive tests. Regression analysis found similar models for both domains. Both models included education, race, anxiety, and delayed recall. Depression was the only unique variable in the memory model, F(5, 17)=4.09, p=.013, R2=.55, and word reading was unique to the cognitive/intellectual functions model, F(5, 15) = 6.91, p = .002, R2 = .70. In contrast, the language domain correlated negatively with four cognitive measures: word reading (r=-.46, p=.038), learning of a word list (r=-.47, p=-.024), delayed recall of a word list (r=-.51, p=.012), and verbal fluency (r=-.49, p=.045). Regression analysis found age, gender, depression, word reading, and delayed recall predict PAOFI language scores, F(5, 15)=6.27, p=.002, R2=.68. These findings suggest mood, demographics, and performance on cognitive tests are related to self-reported cognition. Mood is likely a better predictor of self-reported memory and higher-level cognitive abilities, and cognitive tests may better predict self-reported language abilities.

